# 4-(8-Eth­oxy-2,3-dihydro-1*H*-cyclo­penta­[*c*]quinolin-4-yl)butane-1-peroxol

**DOI:** 10.1107/S1600536810021781

**Published:** 2010-06-16

**Authors:** Jean Fotie, Chris F. Fronczek, Kyle A. Burns, Frank R. Fronczek, Cheryl Bain, D. Scott Bohle, Ferdinand P. Poudeu

**Affiliations:** a Department of Chemistry and Physics, Southeastern Louisiana University, SLU 10878, Hammond, LA 70402-0878, USA; b Department of Chemistry, Louisiana State University, Baton Rouge, LA 70803-1804, USA; c Department of Chemistry, McGill University, Otto Maas Chemistry Building, 801 Sherbrooke Street West, Montreal, Quebec, Canada H3A 2K6; d Department of Chemistry, University of New Orleans, New Orleans, LA 70148, USA

## Abstract

In the title mol­ecule, C_18_H_23_NO_3_, the hydro­per­oxy­butyl substituent is nearly fully extended, with the four torsion angles in the range 170.23 (10)–178.71 (9)°. The O—O distance in the hydro­peroxide group is 1.4690 (13) Å. This group acts as an inter­molecular hydrogen-bond donor to a quinoline N atom. This results in dimeric units about the respective inversion centers, with graph-set notation *R*_2_^2^(18).

## Related literature

For a description of the Cambridge Structural Database, see: Allen (2002 ▶). For graph-set motifs, see: Etter (1990 ▶). For the biological activity of dihydro­quinolines, see: Babiak *et al.* (1999 ▶); Cracknell *et al.* (1998 ▶); Dillard *et al.* (1973 ▶); Fotie *et al.* (2010 ▶); Lockhart *et al.* (2001 ▶); Shah *et al.* (2005 ▶); Takahashi *et al.* (2006 ▶); Thorisson *et al.* (1992 ▶). For related structures, see: Grignon-Dubois *et al.* (1993 ▶); Noland *et al.* (1996 ▶).
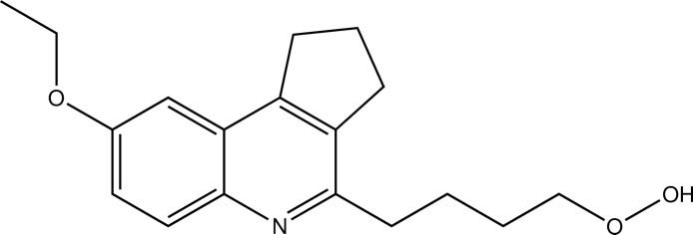


## Experimental

### 

#### Crystal data

C_18_H_23_NO_3_
*M* *_r_* = 301.37
Triclinic, 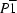

*a* = 8.0113 (2) Å
*b* = 8.5091 (2) Å
*c* = 12.6334 (3) Å
α = 73.605 (1)°
β = 74.936 (1)°
γ = 78.136 (1)°
*V* = 789.63 (3) Å^3^
*Z* = 2
Cu *K*α radiation
μ = 0.69 mm^−1^
*T* = 90 K
0.19 × 0.17 × 0.15 mm

#### Data collection

Bruker APEXII CCD diffractometer
Absorption correction: multi-scan (*SADABS*; Sheldrick, 2004 ▶) *T*_min_ = 0.880, *T*_max_ = 0.904
9369 measured reflections
2798 independent reflections
2400 reflections with *I* > 2σ(*I*)
*R*_int_ = 0.030

#### Refinement

*R*[*F* ^2^ > 2σ(*F* ^2^)] = 0.036
*wR*(*F* ^2^) = 0.110
*S* = 1.08
2798 reflections
202 parameters
H-atom parameters constrained
Δρ_max_ = 0.22 e Å^−3^
Δρ_min_ = −0.27 e Å^−3^

### 

Data collection: *APEX2* (Bruker, 2006 ▶); cell refinement: *SAINT* (Bruker, 2006 ▶); data reduction: *SAINT*; program(s) used to solve structure: *SHELXS97* (Sheldrick, 2008 ▶); program(s) used to refine structure: *SHELXL97* (Sheldrick, 2008 ▶); molecular graphics: *ORTEP-3 for Windows* (Farrugia, 1997 ▶); software used to prepare material for publication: *SHELXTL* (Sheldrick, 2008 ▶).

## Supplementary Material

Crystal structure: contains datablocks global, I. DOI: 10.1107/S1600536810021781/fj2311sup1.cif

Structure factors: contains datablocks I. DOI: 10.1107/S1600536810021781/fj2311Isup2.hkl

Additional supplementary materials:  crystallographic information; 3D view; checkCIF report

## Figures and Tables

**Table 1:** Hydrogen-bond geometry (Å, °)

*D*—H⋯*A*	*D*—H	H⋯*A*	*D*⋯*A*	*D*—H⋯*A*
O2—H2⋯N1^i^	0.84	1.93	2.7466 (14)	165

Symmetry code: (i) 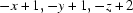.

**Fig. 1.**
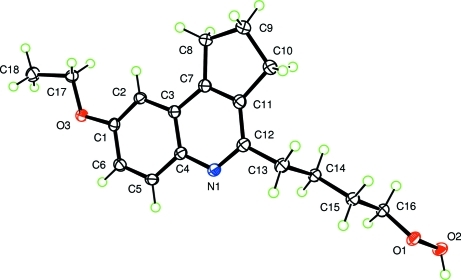
Ellipsoids at the 50% level, with H atoms having arbitrary radius.

**Fig. 2.**
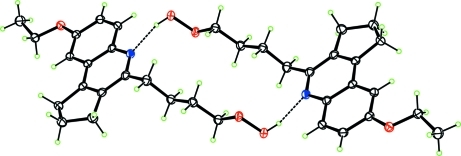
The hydrogen-bonded dimer, with graph set R22(18).

## supplementary crystallographic information

### Comment

Dihydroquinolines are mainly known for their antioxidant activity (Thorisson *et al.*, 1992, Lockhart *et al.*, 2001) although they have also been reported to possess anti-inflammatory (Dillard *et al.*, 1973), fungicidal (Cracknell *et al.*, 1998), antiatherosclerotic (Babiak *et al.*, 1999), and hormone receptor modulator (Takahashi *et al.*, 2006) properties. Furthermore, 6-ethoxy-1,2-dihydro-2,2,4-trimethylquinoline, also known as ethoxyquin, is a FDA approved antioxidant commonly used as a preservative in the food processing industry (Shah *et al.*, 2005). We have recently reported some dihydroquinoline derivatives with outstanding antitrypanosomal activity (Fotie *et al.*, 2010). In our effort to optimize the trypanocidal activity of this family of compound, we have synthesized the title compound, an unusual hydroperoxybutylquinoline derivative. Here we are reporting the characterization of that compound using ^1^H– and ^13^C-NMR spectroscopy, mass spectrometry, and single-crystal diffraction.

The molecular structure of the title compound is illustrated in Fig. 1. The 10-atom quinoline ring system is essentially planar, with mean deviation 0.009 Å and maximum deviation 0.017 (1) Å for both N1 and C11. The five-membered ring has the envelope conformation, with C9 at the flap position, 0.340 (2) Å out of the quinoline plane. The hydroperoxybutyl chain is extended, with torsion angle magnitudes in the range 170.23 (10) to 178.71 (9)°, and the best plane of its four C and two O atoms is approximately perpendicular to the quinoline plane, forming a dihedral angle of 89.53 (3)°. The hydroperoxy O—O distance, 1.4690 (13) Å agrees well with literature values for this group. The mean value of the 135 such distances in the Cambridge Structural Database (version 5.31, Nov. 2009; Allen 2002), after rejecting eight outliers, is 1.462 Å.

The hydroperoxide donates an intermolecular hydrogen bond to quinoline N1, with O···N distance 2.7466 (14) Å, forming discrete dimers having graph set (Etter, 1990) *R*^2^_2_(18) about inversion centers, as illustrated in Fig. 2.

### Experimental

The title compound was prepared by heating to reflux for three days, a mixture of *p*-phenitidine (500 mg, 3.6 mmol) and cyclopentanone (10 ml, large excess) in the presence of catalytic amounts of iodine (93 mg) and benzoyl peroxide (8.8 mg). After appropriate work-up, and purification on a silica gel column, crystals were carefully grown at room temperature, in a mixture of hexanes-dichloromethane, over the course of a week.

Mp: 131.3 - 131.6 °C. The melting point was recorded on a MEL-TEMP ELECTROTHERMAL digital melting point apparatus, and is not corrected.

ESIMS *m/z* (%): 316 (90) [*M* + CH_3_]^+^, 302 (43) [*M* + H]^+^, 286 (100) [*M* -16]^+^, 284 (94) [*M* - H_2_O]^+^. These fragment ions are consistent with a molecular formula of C_18_H_23_NO_3_. The ESIMS spectrum was recorded on a Finnigan LCQDUO spectrometer.

NMR data were collected on a Bruker AC 300 Spectrometer. ^1^H-NMR (300 MHz, CDCl_3_) δ: 1.47 (3*H*, t, J = 6.7 Hz), 1.68 (2*H*, m), 1.85 (2*H*, m), 2.23 (2*H*, m), 3.04 (4*H*, t, J = 7.9 Hz), 3.13 (2*H*, t, J = 7.3 Hz), 4.10 (2*H*, t, 6.7 Hz), 4.15 (2*H*, q, J = 6.7 Hz), 6.90 (1*H*, d, J = 2.4 Hz), 7.23 (1*H*, dd, J = 9.2 Hz and 2,4 Hz), 7.83 (1*H*, d, 9.2 Hz), 13.6 (1*H*, brs). ^13^C-NMR (75 MHz, CDCl_3_) δ: 14.9, 24.0, 25.0, 25.9, 31.4, 31.5, 35.0, 63.8, 103.0, 121.1, 125.9, 129.2, 136.0, 141.6, 149.7, 155.9, 156.6. 162.3.

### Refinement

H atoms on C were placed in idealized positions with C—H distances 0.95 - 1.00 Å and thereafter treated as riding. The OH H atom was located from a difference map in the expected circle. Torsional parameters were refined for the methyl and hydroperoxy OH groups. *U*_iso_ for H were assigned as 1.2 times *U*_eq_ of the attached atoms (1.5 for methyl and OH).

### Crystal data

**Table:**

C_18_H_23_NO_3_	*Z* = 2
*M**_r_* = 301.37	*F*(000) = 324
Triclinic, *P*1	*D*_x_ = 1.268 Mg m^−^^3^
Hall symbol: -P 1	Cu *K*α radiation, λ = 1.54178 Å
*a* = 8.0113 (2) Å	Cell parameters from 4518 reflections
*b* = 8.5091 (2) Å	θ = 3.7–68.3°
*c* = 12.6334 (3) Å	µ = 0.69 mm^−^^1^
α = 73.605 (1)°	*T* = 90 K
β = 74.936 (1)°	Prism, colourless
γ = 78.136 (1)°	0.19 × 0.17 × 0.15 mm
*V* = 789.63 (3) Å^3^	

### Data collection

**Table:**

Bruker APEXII CCD diffractometer	2798 independent reflections
Radiation source: fine-focus sealed tube	2400 reflections with *I* > 2σ(*I*)
graphite	*R*_int_ = 0.030
φ and ω scans	θ_max_ = 68.8°, θ_min_ = 3.7°
Absorption correction: multi-scan (*SADABS*; Sheldrick, 2004)	*h* = −9→9
*T*_min_ = 0.880, *T*_max_ = 0.904	*k* = −9→10
9369 measured reflections	*l* = −15→15

### Refinement

**Table:**

Refinement on *F*^2^	Secondary atom site location: difference Fourier map
Least-squares matrix: full	Hydrogen site location: inferred from neighbouring sites
*R*[*F*^2^ > 2σ(*F*^2^)] = 0.036	H-atom parameters constrained
*wR*(*F*^2^) = 0.110	*w* = 1/[σ^2^(*F*_o_^2^) + (0.0626*P*)^2^ + 0.1754*P*] where *P* = (*F*_o_^2^ + 2*F*_c_^2^)/3
*S* = 1.08	(Δ/σ)_max_ < 0.001
2798 reflections	Δρ_max_ = 0.22 e Å^−^^3^
202 parameters	Δρ_min_ = −0.27 e Å^−^^3^
0 restraints	Extinction correction: *SHELXL97* (Sheldrick, 2008), Fc^*^=kFc[1+0.001xFc^2^λ^3^/sin(2θ)]^-1/4^
Primary atom site location: structure-invariant direct methods	Extinction coefficient: 0.0023 (7)

### Special details

**Table:**

Geometry. All e.s.d.'s (except the e.s.d. in the dihedral angle between two l.s. planes) are estimated using the full covariance matrix. The cell e.s.d.'s are taken into account individually in the estimation of e.s.d.'s in distances, angles and torsion angles; correlations between e.s.d.'s in cell parameters are only used when they are defined by crystal symmetry. An approximate (isotropic) treatment of cell e.s.d.'s is used for estimating e.s.d.'s involving l.s. planes.
Refinement. Refinement of *F*^2^ against ALL reflections. The weighted *R*-factor *wR* and goodness of fit *S* are based on *F*^2^, conventional *R*-factors *R* are based on *F*, with *F* set to zero for negative *F*^2^. The threshold expression of *F*^2^ > σ(*F*^2^) is used only for calculating *R*-factors(gt) *etc*. and is not relevant to the choice of reflections for refinement. *R*-factors based on *F*^2^ are statistically about twice as large as those based on *F*, and *R*- factors based on ALL data will be even larger.

### Fractional atomic coordinates and isotropic or equivalent isotropic displacement parameters (Å^2^)

**Table:**

	*x*	*y*	*z*	*U*_iso_*/*U*_eq_	
O1	0.54940 (12)	0.44045 (11)	1.21141 (7)	0.0202 (2)	
O2	0.43514 (12)	0.35072 (12)	1.31138 (8)	0.0233 (3)	
H2	0.3429	0.4140	1.3292	0.035*	
O3	0.98882 (12)	0.17854 (11)	0.27498 (8)	0.0201 (2)	
N1	0.84064 (14)	0.40032 (13)	0.66551 (9)	0.0180 (3)	
C1	0.96537 (17)	0.22269 (16)	0.37459 (11)	0.0177 (3)	
C2	1.08402 (17)	0.17863 (16)	0.44285 (11)	0.0175 (3)	
H2A	1.1914	0.1104	0.4233	0.021*	
C3	1.04475 (17)	0.23589 (15)	0.54275 (11)	0.0166 (3)	
C4	0.88489 (17)	0.33756 (16)	0.57160 (11)	0.0171 (3)	
C5	0.76525 (17)	0.37924 (16)	0.49902 (11)	0.0184 (3)	
H5	0.6573	0.4473	0.5172	0.022*	
C6	0.80419 (17)	0.32234 (16)	0.40359 (11)	0.0198 (3)	
H6	0.7225	0.3498	0.3562	0.024*	
C7	1.15889 (17)	0.19861 (16)	0.61825 (11)	0.0173 (3)	
C8	1.33845 (17)	0.09920 (17)	0.60806 (11)	0.0203 (3)	
H8A	1.4123	0.1362	0.5321	0.024*	
H8B	1.3323	−0.0203	0.6226	0.024*	
C9	1.40947 (18)	0.13488 (18)	0.69997 (12)	0.0235 (3)	
H9A	1.4726	0.0323	0.7404	0.028*	
H9B	1.4909	0.2177	0.6656	0.028*	
C10	1.25038 (18)	0.20199 (18)	0.78213 (12)	0.0229 (3)	
H10A	1.2155	0.1143	0.8507	0.027*	
H10B	1.2753	0.2956	0.8047	0.027*	
C11	1.11048 (17)	0.25808 (16)	0.71348 (11)	0.0188 (3)	
C12	0.95014 (17)	0.36144 (16)	0.73497 (11)	0.0184 (3)	
C13	0.89624 (18)	0.43704 (17)	0.83518 (11)	0.0209 (3)	
H13A	0.8268	0.5471	0.8139	0.025*	
H13B	1.0025	0.4540	0.8543	0.025*	
C14	0.78889 (17)	0.33287 (16)	0.94029 (11)	0.0186 (3)	
H14A	0.6851	0.3105	0.9213	0.022*	
H14B	0.8601	0.2254	0.9657	0.022*	
C15	0.73048 (17)	0.42275 (16)	1.03551 (11)	0.0190 (3)	
H15A	0.8351	0.4403	1.0561	0.023*	
H15B	0.6655	0.5329	1.0079	0.023*	
C16	0.61601 (18)	0.32967 (16)	1.13985 (11)	0.0194 (3)	
H16A	0.5194	0.2962	1.1196	0.023*	
H16B	0.6850	0.2291	1.1779	0.023*	
C17	1.15683 (18)	0.09386 (16)	0.23380 (11)	0.0201 (3)	
H17A	1.1735	−0.0203	0.2812	0.024*	
H17B	1.2503	0.1526	0.2357	0.024*	
C18	1.1631 (2)	0.09065 (18)	0.11403 (12)	0.0256 (3)	
H18A	1.0682	0.0347	0.1132	0.038*	
H18B	1.2755	0.0309	0.0835	0.038*	
H18C	1.1496	0.2043	0.0676	0.038*	

### Atomic displacement parameters (Å^2^)

**Table:**

	*U*^11^	*U*^22^	*U*^33^	*U*^12^	*U*^13^	*U*^23^
O1	0.0200 (5)	0.0214 (5)	0.0171 (5)	−0.0013 (4)	0.0010 (4)	−0.0070 (4)
O2	0.0197 (5)	0.0255 (5)	0.0176 (5)	0.0011 (4)	0.0027 (4)	−0.0033 (4)
O3	0.0201 (5)	0.0229 (5)	0.0180 (5)	0.0001 (4)	−0.0048 (4)	−0.0076 (4)
N1	0.0179 (6)	0.0168 (6)	0.0179 (6)	−0.0021 (4)	−0.0017 (4)	−0.0044 (4)
C1	0.0208 (7)	0.0161 (7)	0.0153 (6)	−0.0040 (5)	−0.0026 (5)	−0.0027 (5)
C2	0.0164 (7)	0.0150 (6)	0.0183 (7)	−0.0013 (5)	−0.0018 (5)	−0.0023 (5)
C3	0.0171 (7)	0.0141 (6)	0.0166 (7)	−0.0038 (5)	−0.0023 (5)	−0.0006 (5)
C4	0.0181 (7)	0.0141 (6)	0.0168 (7)	−0.0033 (5)	−0.0004 (5)	−0.0022 (5)
C5	0.0151 (7)	0.0165 (7)	0.0210 (7)	0.0002 (5)	−0.0027 (5)	−0.0029 (5)
C6	0.0187 (7)	0.0193 (7)	0.0199 (7)	−0.0030 (5)	−0.0051 (5)	−0.0017 (5)
C7	0.0173 (7)	0.0148 (6)	0.0175 (7)	−0.0039 (5)	−0.0023 (5)	−0.0003 (5)
C8	0.0176 (7)	0.0211 (7)	0.0201 (7)	0.0016 (5)	−0.0045 (5)	−0.0045 (5)
C9	0.0183 (7)	0.0282 (8)	0.0235 (7)	−0.0008 (6)	−0.0065 (6)	−0.0056 (6)
C10	0.0222 (7)	0.0259 (8)	0.0219 (7)	−0.0011 (6)	−0.0075 (6)	−0.0071 (6)
C11	0.0194 (7)	0.0180 (7)	0.0175 (7)	−0.0052 (5)	−0.0029 (5)	−0.0012 (5)
C12	0.0193 (7)	0.0164 (7)	0.0182 (7)	−0.0043 (5)	−0.0017 (5)	−0.0032 (5)
C13	0.0215 (7)	0.0203 (7)	0.0212 (7)	−0.0037 (5)	−0.0020 (6)	−0.0075 (6)
C14	0.0194 (7)	0.0188 (7)	0.0182 (7)	−0.0005 (5)	−0.0043 (5)	−0.0067 (5)
C15	0.0181 (7)	0.0203 (7)	0.0192 (7)	−0.0006 (5)	−0.0041 (5)	−0.0072 (5)
C16	0.0213 (7)	0.0195 (7)	0.0179 (7)	0.0005 (5)	−0.0043 (5)	−0.0075 (5)
C17	0.0214 (7)	0.0180 (7)	0.0197 (7)	−0.0001 (5)	−0.0029 (5)	−0.0058 (5)
C18	0.0307 (8)	0.0246 (8)	0.0226 (7)	−0.0005 (6)	−0.0046 (6)	−0.0106 (6)

### Geometric parameters (Å, °)

**Table:**

O1—C16	1.4193 (15)	C9—H9B	0.9900
O1—O2	1.4690 (13)	C10—C11	1.5125 (18)
O2—H2	0.8400	C10—H10A	0.9900
O3—C1	1.3693 (15)	C10—H10B	0.9900
O3—C17	1.4319 (16)	C11—C12	1.4094 (19)
N1—C12	1.3288 (17)	C12—C13	1.5048 (18)
N1—C4	1.3709 (17)	C13—C14	1.5310 (18)
C1—C2	1.3729 (18)	C13—H13A	0.9900
C1—C6	1.4142 (19)	C13—H13B	0.9900
C2—C3	1.4166 (18)	C14—C15	1.5266 (17)
C2—H2A	0.9500	C14—H14A	0.9900
C3—C4	1.4147 (18)	C14—H14B	0.9900
C3—C7	1.4176 (18)	C15—C16	1.5122 (18)
C4—C5	1.4208 (18)	C15—H15A	0.9900
C5—C6	1.3628 (19)	C15—H15B	0.9900
C5—H5	0.9500	C16—H16A	0.9900
C6—H6	0.9500	C16—H16B	0.9900
C7—C11	1.3691 (19)	C17—C18	1.5087 (18)
C7—C8	1.5054 (18)	C17—H17A	0.9900
C8—C9	1.5420 (19)	C17—H17B	0.9900
C8—H8A	0.9900	C18—H18A	0.9800
C8—H8B	0.9900	C18—H18B	0.9800
C9—C10	1.5413 (19)	C18—H18C	0.9800
C9—H9A	0.9900		
			
C16—O1—O2	105.80 (9)	C7—C11—C12	120.33 (12)
O1—O2—H2	109.5	C7—C11—C10	111.14 (12)
C1—O3—C17	117.05 (10)	C12—C11—C10	128.50 (12)
C12—N1—C4	119.06 (11)	N1—C12—C11	121.27 (12)
O3—C1—C2	125.10 (12)	N1—C12—C13	116.62 (11)
O3—C1—C6	114.06 (11)	C11—C12—C13	122.09 (12)
C2—C1—C6	120.84 (12)	C12—C13—C14	114.07 (11)
C1—C2—C3	119.32 (12)	C12—C13—H13A	108.7
C1—C2—H2A	120.3	C14—C13—H13A	108.7
C3—C2—H2A	120.3	C12—C13—H13B	108.7
C4—C3—C2	120.22 (12)	C14—C13—H13B	108.7
C4—C3—C7	116.10 (12)	H13A—C13—H13B	107.6
C2—C3—C7	123.68 (12)	C15—C14—C13	110.73 (11)
N1—C4—C3	123.16 (12)	C15—C14—H14A	109.5
N1—C4—C5	118.20 (12)	C13—C14—H14A	109.5
C3—C4—C5	118.63 (12)	C15—C14—H14B	109.5
C6—C5—C4	120.53 (12)	C13—C14—H14B	109.5
C6—C5—H5	119.7	H14A—C14—H14B	108.1
C4—C5—H5	119.7	C16—C15—C14	113.20 (11)
C5—C6—C1	120.44 (12)	C16—C15—H15A	108.9
C5—C6—H6	119.8	C14—C15—H15A	108.9
C1—C6—H6	119.8	C16—C15—H15B	108.9
C11—C7—C3	120.02 (12)	C14—C15—H15B	108.9
C11—C7—C8	111.83 (11)	H15A—C15—H15B	107.8
C3—C7—C8	128.14 (12)	O1—C16—C15	106.06 (11)
C7—C8—C9	103.20 (11)	O1—C16—H16A	110.5
C7—C8—H8A	111.1	C15—C16—H16A	110.5
C9—C8—H8A	111.1	O1—C16—H16B	110.5
C7—C8—H8B	111.1	C15—C16—H16B	110.5
C9—C8—H8B	111.1	H16A—C16—H16B	108.7
H8A—C8—H8B	109.1	O3—C17—C18	107.10 (11)
C10—C9—C8	106.77 (11)	O3—C17—H17A	110.3
C10—C9—H9A	110.4	C18—C17—H17A	110.3
C8—C9—H9A	110.4	O3—C17—H17B	110.3
C10—C9—H9B	110.4	C18—C17—H17B	110.3
C8—C9—H9B	110.4	H17A—C17—H17B	108.6
H9A—C9—H9B	108.6	C17—C18—H18A	109.5
C11—C10—C9	103.12 (11)	C17—C18—H18B	109.5
C11—C10—H10A	111.1	H18A—C18—H18B	109.5
C9—C10—H10A	111.1	C17—C18—H18C	109.5
C11—C10—H10B	111.1	H18A—C18—H18C	109.5
C9—C10—H10B	111.1	H18B—C18—H18C	109.5
H10A—C10—H10B	109.1		
			
C17—O3—C1—C2	−6.48 (18)	C3—C7—C8—C9	168.11 (13)
C17—O3—C1—C6	173.01 (11)	C7—C8—C9—C10	18.58 (14)
O3—C1—C2—C3	178.62 (11)	C8—C9—C10—C11	−19.42 (14)
C6—C1—C2—C3	−0.8 (2)	C3—C7—C11—C12	−2.3 (2)
C1—C2—C3—C4	−0.07 (19)	C8—C7—C11—C12	176.70 (12)
C1—C2—C3—C7	−179.45 (12)	C3—C7—C11—C10	179.39 (11)
C12—N1—C4—C3	−1.71 (19)	C8—C7—C11—C10	−1.59 (16)
C12—N1—C4—C5	179.37 (11)	C9—C10—C11—C7	13.27 (15)
C2—C3—C4—N1	−178.41 (11)	C9—C10—C11—C12	−164.84 (13)
C7—C3—C4—N1	1.02 (19)	C4—N1—C12—C11	0.34 (19)
C2—C3—C4—C5	0.51 (19)	C4—N1—C12—C13	178.70 (11)
C7—C3—C4—C5	179.94 (11)	C7—C11—C12—N1	1.7 (2)
N1—C4—C5—C6	178.92 (11)	C10—C11—C12—N1	179.63 (12)
C3—C4—C5—C6	−0.05 (19)	C7—C11—C12—C13	−176.59 (12)
C4—C5—C6—C1	−0.8 (2)	C10—C11—C12—C13	1.4 (2)
O3—C1—C6—C5	−178.21 (11)	N1—C12—C13—C14	89.79 (14)
C2—C1—C6—C5	1.3 (2)	C11—C12—C13—C14	−91.87 (15)
C4—C3—C7—C11	1.01 (19)	C12—C13—C14—C15	−176.62 (11)
C2—C3—C7—C11	−179.59 (12)	C13—C14—C15—C16	177.09 (10)
C4—C3—C7—C8	−177.84 (12)	O2—O1—C16—C15	178.71 (9)
C2—C3—C7—C8	1.6 (2)	C14—C15—C16—O1	−170.23 (10)
C11—C7—C8—C9	−10.82 (15)	C1—O3—C17—C18	−168.40 (11)

### Hydrogen-bond geometry (Å, °)

**Table:**

*D*—H···*A*	*D*—H	H···*A*	*D*···*A*	*D*—H···*A*
O2—H2···N1^i^	0.84	1.93	2.7466 (14)	165

Symmetry codes: (i) −*x*+1, −*y*+1, −*z*+2.
